# Reproductive outcomes in adolescents who had a previous birth or an induced abortion compared to adolescents' first pregnancies

**DOI:** 10.1186/1471-2393-8-4

**Published:** 2008-01-31

**Authors:** Birgit Reime, Beate A Schücking, Paul Wenzlaff

**Affiliations:** 1 Faculty of Nursing and Healthcare, University of Applied Sciences of the Saarland, Goebenstr. 40, 66117 Saarbrücken, Germany; 2 Fachbereich Gesundheitswissenschaften, University of Osnabrück, Albrechtstr 28, 49046 Osnabrück, Germany; 3 Centre for Quality Management, Physicians' Chamber of Lower Saxony, Berliner Allee 20, 30175 Hanover, Germany

## Abstract

**Background:**

Recently, attention has been focused on subsequent pregnancies among teenage mothers. Previous studies that compared the reproductive outcomes of teenage nulliparae and multiparae often did not consider the adolescents' reproductive histories. Thus, the authors compared the risks for adverse reproductive outcomes of adolescent nulliparae to teenagers who either have had an induced abortion or a previous birth.

**Methods:**

In this retrospective cohort study we used perinatal data prospectively collected by obstetricians and midwives from 1990–1999 (participation rate 87–98% of all hospitals) in Lower Saxony, Germany. From the 9742 eligible births among adolescents, women with multiple births, >1 previous pregnancies, or a previous spontaneous miscarriage were deleted and 8857 women <19 years remained. Of these 8857 women, 7845 were nulliparous, 801 had one previous birth, and 211 had one previous induced abortion. The outcomes were stillbirths, neonatal mortality, perinatal mortality, preterm births, and very low birthweight. Bivariate and multivariable logistic regression models were conducted.

**Results:**

In bivariate logistic regression analyses, compared to nulliparous teenagers, adolescents with a previous birth had higher risks for perinatal [OR = 2.08, CI = 1.11,3.89] and neonatal [OR = 4.31, CI = 1.77,10.52] mortality and adolescents with a previous abortion had higher risks for stillbirths [OR = 3.31, CI = 1.01,10.88] and preterm births [OR = 2.21, CI = 1.07,4.58]. After adjusting for maternal nationality, partner status, smoking, prenatal care and pre-pregnancy BMI, adolescents with a previous birth were at higher risk for perinatal [OR = 2.35, CI = 1.14,4.86] and neonatal mortality [OR = 4.70, CI = 1.60,13.81] and adolescents with a previous abortion had a higher risk for very low birthweight infants [OR = 2.74, CI = 1.06,7.09] than nulliparous teenagers.

**Conclusion:**

The results suggest that teenagers who give birth twice as adolescents have worse outcomes in their second pregnancy compared to those teenagers who are giving birth for the first time. The prevention of the second pregnancy during adolescence is an important public health objective and should be addressed by health care providers who attend the first birth or the abortion and the follow-up care. Also, health care workers should attempt to improve the pregnancy outcomes of subsequent teenage pregnancies by addressing modifiable risk factors, for example, supporting smoking cessation and utilization of prenatal care.

## Background

Teenage pregnancy is a significant public health issue. Giving birth during adolescence is strongly associated with adverse living conditions in later life [1]. Approximately 1.25 million teenagers become pregnant each year in the 28 OECD (Organisation for Economic Co-operation and Development) nations [2]. Of those, about half a million pregnancies will be terminated and approximately three quarters of a million teenagers will become mothers. In 2003, The Netherlands, Sweden, Denmark, Finland and Slovenia had the lowest adolescent birth rates in Europe (6/1,000) while the United Kingdom (27/1,000) was characterized by the highest rates [3].

In Germany, the proportion of mothers between 10 and 18 years of age rose from 0.9% in 2000 to 1.0% in 2006 (19/1,000 women of the same age) [4]. Among women who had an induced abortion the proportion of adolescent women rose from 4.7% in 2000 to 5.5% in 2006 (17/1,000 to 19/1,000). In the western federal state Lower Saxony between 2000 and 2006 the rate of live births ranged from 22 to 18 while the rates in Berlin and in the eastern federal states were 20–70% higher during this period of time. The same regional pattern can be observed regarding the rates of induced abortions [4].

Recently, attention has been focused on subsequent pregnancies among teenage mothers. The likelihood of a second birth among adolescent mothers is much greater than the likelihood of a first birth among teen females who have not had a child yet. For example, in the United States in 2001, there were 35.7 births per 1000 females aged 15 to 19 years who never had a birth compared to 175.1 births per 1000 females aged 15 to 19 years who previously had one birth [5]. Accordingly, twenty percent of teen births occurred to young women who had been mothers already. In a representative sample of adolescent mothers in the US about two thirds reported that the second pregnancy was not intended [6]. In Germany, among those teenagers who had an abortion in 2006, 2.8% reported to have a child already [4].

A small number of studies have examined the relationship between parity and reproductive outcomes among teenagers. Cross-sectional studies suggest a lower risk for low birthweight [7] and for neonatal, postneonatal and infant mortality [7-9] in the first pregnancy of adolescent women. However, the results of longitudinal studies are similar to studies on adult women populations [10], and indicate that higher rates of low birthweight infants [11] and intrauterine growth retardation [12] are associated with teenagers' first birth compared to their second.

Previous studies on parity and reproductive outcomes in adolescents had several important limitations. Cross-sectional studies based on the linking of birth and death records could not access relevant confounders [7,9]. Longitudinal studies that followed individuals over time had samples that didn't reflect the general population, were based on small geographical areas and/or lacked statistical power because of a small sample size [12-15]. Only one longitudinal study considered the adolescents' obstetric history regarding abortions and miscarriages [13]. The objective of this study was to compare the perinatal outcomes (rates of stillbirths, neonatal and perinatal mortality, preterm birth and very low birthweight) of nulliparous teenagers and teenagers who previously had an induced abortion or a live- or stillbirth after adjustment for potential confounders (maternal nationality, partner status, smoking, prenatal care and pre-pregnancy BMI).

## Methods

### Study design and setting

We examined the relationship between reproductive history and reproductive outcomes among nulliparous adolescents and adolescents who had a previous abortion or a previous birth using routinely collected perinatal data. In most federal states of Germany, pregnancy and delivery-related data are maintained in a central perinatal registry. Our study is based on the Perinatal Surveys of the years 1990–1999 in Lower Saxony (742,031 cases). Prospectively during pregnancy, midwives and obstetricians collect data on maternal socio-demographic background, maternal health and behaviour, obstetrical care and interventions during pregnancy in a "mother's passport". The mother receives this document at the first prenatal visit during her first pregnancy and the data on all of her pregnancies are recorded in this passport. This refers to induced abortions, and miscarriages or stillbirths as well as to live-births. The mother has to bring this passport with her when she gives birth in hospital to inform the staff on her reproductive history. Information on interventions during birth and infant outcomes up to seven days post partum are collected after birth. Hospital staff electronically submit registry data derived from the mother's passports and delivery records to the Centre for Quality Management of the Physicians' Chamber of Lower Saxony. Because these data are collected routinely on an anonymous basis (comparable to vital statistics) in Germany informed consent is not required for this procedure. All live-births and stillborn infants with a birthweight of >499 grams (until April 1^st ^1994: >999 grams) are included in the registry. The data are restricted to hospital births that make up more than 98 percent of all births in Lower Saxony. The participation rate of the hospitals in Lower Saxony was 87 percent in 1990, and steadily increased to 98 percent in 1999. The non-participating hospitals stated to have insufficient resources for participation. However, they did not differ from the participating hospitals in terms of health outcomes, the type of hospital, number of births per year, or any other known characteristic [16].

Lower Saxony is a large federal state in the northwest of Germany (Hanover is the capital) that consists of several large cities as well as vast rural areas. The population in Lower Saxony is rather homogeneous in terms of ethnicity. During the 1990's, the proportion of female migrant adolescents between 15 and 20 years of age ranged from 6.3 percent to 8.1 percent [17].

### Study sample

In this sample there are 7845 nulliparous teenagers, 801 teenagers with one previous live- or stillbirth, and 211 teenagers who had one previous abortion. We restricted our sample to these groups because we attempted to avoid effects resulting from unmeasured confounders that are associated with higher order births and heterogeneous reproductive histories. Because we used anonymised data from a perinatal registry, ethic approval was not required.

### Variables

#### Reproductive history

Adolescents with no prior pregnancies recorded in the mother's passport were defined as nulliparous. Teenagers with a previous pregnancy were divided into women with a previous live- or stillbirth and those with a history of abortion.

#### Outcomes: Definitions and denominators

In Germany, neonatal mortality is defined as the death of a live born infant (showing heartbeat, lung breathing, and/or pulsation of the cord) of any weight occurring up to 7 days post partum. Stillbirth is defined as a birth of an infant without live-signs weighing more than 999 grams (until March 31st 1994) or more than 499 grams (from April 1st 1994 onward). Perinatal mortality is defined as the sum of stillbirths and neonatal mortality and was obtained by summing the number of infants coded as stillbirths and as neonatal deaths. For all three variables the denominator was all births. Preterm birth is defined as births occurring before 259 days (37+0 weeks) of gestation, very low birthweight (VLBW) is defined as infants weighing <1500 grams. The denominator of the last two outcomes was all live births.

#### Potential Confounders

Nationality was coded as German national or migrant, and partner status was coded as living single or with a partner. The variable smoking during pregnancy was collapsed into binary format (0 versus any cigarettes). Inadequate prenatal care (yes/no) was determined by the Adequacy of Prenatal Care Utilisation (APNCU) Index [18], which combines information about the time of initiation of prenatal care and the total number of prenatal visits, adjusted for gestational age at birth. Less than 50 percent of the recommended number of prenatal visits for a given gestational age and initiation of prenatal care after the first trimester is defined as "inadequate care" [18]. BMI was calculated as pre-pregnancy body weight (in kilograms) divided by the square of height (in meters) and was entered as a continuous variable.

### Statistical analyses

Using SPSS 12.0, we performed chi-square- and t-tests to study the relationship between reproductive history and the potential confounders. Crude odds ratios, including 95 percent-confidence intervals, were computed to examine the associations between the adolescents' reproductive history (referent group: nulliparous teenagers) and the pregnancy outcomes. In multivariable logistic regression models, these associations were adjusted for nationality, partner status, smoking, prenatal care, and BMI. Diagnostic analyses were performed on the logistic models as recommended by Hosmer and Lemeshow (1989) [19]. We defined p < 0.05 as statistically significant. In this retrospective cohort study there were missings related to exposures collected during prenatal visits and related to outcomes collected after birth. Cases with missing values were deleted.

## Results

### Participants

There were 9742 births among teenagers aged 13–18 years. After exclusion of multiple births (n = 130), ≥ 2 previous pregnancies (n = 237) and a previous spontaneous miscarriage (as a proxy for a potential genetic disorder) (n = 377), 8857 young women remained. Of these 8857 women, 7845 were nulliparous, 801 were teenagers with one previous live- or stillbirth, and 211 had one previous abortion.

### Demographic characteristics

The majority (96.1 percent) of the adolescents were aged between 16 and 18 years and more than one third (37.2 percent) were migrants. Among migrants, the majority were from the Middle East (51.2 percent), Mediterranean countries (23.2 percent) and Eastern Europe (17.4 percent).

Table 1 contains the results of the analyses of the relationship between reproductive history and potential confounders. Among teenagers who had a previous abortion, the proportion of smokers (46.0 percent) and single parents (40.1 percent) was higher than among the two other groups (p < 0.001). About two thirds of adolescents with a previous birth compared to 13.7 percent of women who previously had an abortion and 34.8 percent of nulliparous women were of migrant nationality (p < 0.001). Inadequate prenatal care was observed mostly among adolescents with a previous birth (42.6 percent), followed by nulliparous adolescents (32.4 percent) and adolescents with a history of abortion (29.4 percent, p < 0.001).

**Table 1:** Maternal characteristics among teenagers with different reproductive histories (chi-square tests and t-tests).

	Nulliparous	Previous birth	History of abortion	P-value
Nationality				<0.001
Migrant n (%)	2730 (34.8)	535 (66.8)	29 (13.7)	
German n (%)	5115 (65.2)	266 (33.2)	182 (86.3)	
Partner status				<0.001
Single parent n (%)	2562 (34.0)	112 (14.2)	81 (40.1)	
Cohabiting n (%)	4980 (66.0)	678 (85.8)	121 (59.9)	
Smoking				<0.001
Yes n (%)	2275 (30.2)	187 (24.1)	93 (46.0)	
No n (%)	5256 (69.8)	589 (75.9)	109 (54.0)	
Prenatal care				<0.001
Inadequate n (%)	2541 (32.4)	341 (42.6)	62 (29.4)	
Adequate n (%)	5304 (67.6)	460 (57.4)	149 (70.6)	
BMI Mean (SD)	22.8 (3.6)	23.5 (4.0)	23.0 (3.6)	<0.001

### Crude analyses

Compared to nulliparous adolescents, adolescents with a previous birth were at higher risk for perinatal [OR = 2.08, CI 1.11, 3.89] and neonatal mortality [OR = 4.31, CI 1.77, 10.52]. Teenagers who previously had an abortion had a 3.3-fold [95 percent CI = 1.01, 10.88] higher risk for a stillbirth and a 2.2-fold [95 percent CI = 1.07, 4.58] higher risk for a preterm born infant than nulliparous adolescents (Table 2).

**Table 2:** Results from bivariate and multivariable regression models regarding the associations between reproductive history and outcomes.

	n (%)	Crude OR (95%-CI)	AOR* (95%-CI)
**Perinatal mortality**			
Nulliparous	57 (0.7)	1.0	1.0
Previous birth	12 (1.5)	**2.08 [1.11, 3.89]**	**2.35 [1.14, 4.86]**
History of abortion	4 (1.9)	2.64 [0.95, 7.35]	1.83 [0.43, 7.68]
**Neonatal mortality**			
Nulliparous	16 (0.2)	1.0	1.0
Previous birth	7 (0.9)	**4.31 [1.77, 10.5]**	**4.70 [1.60, 13.8]**
History of abortion	1 (0.5)	2.33 [0.31, 17.7]	4.64 [0.58, 37.5]
**Stillbirth**			
Nulliparous	36 (0.5)	1.0	1.0
Previous birth	5 (0.6)	1.15 [0.41, 3.26]	1.09 [0.32, 3.71]
History of abortion	3 (1.5)	**3.31 [1.01, 10.9]**	1.23 [0.17, 9.15]
**Very low birthweight**			
Nulliparous	110 (1.4)	1.0	1.0
Previous birth	8 (1.0)	0.62 [0.27, 1.43]	0.53 [0.21, 1.34]
History of abortion	6 (2.8)	2.02 [0.81, 5.02]	**2.74 [1.06, 7.09]**
**Preterm birth**			
Nulliparous	275 (3.5)	1.0	1.0
Previous birth	32 (4.0)	1.13 [0.66, 1.88]	1.08 [0.58, 2.02]
History of abortion	17 (7.7)	**2.21 [1.07, 4.58]**	1.90 [0.77, 4.69]

Acknowledgment: Crude OR = crude odds ratios, AOR = adjusted odds ratios, 95%-CI = 95% confidence intervals.

*Adjusted for nationality, partner status, smoking, inadequate prenatal care and BMI.

### Multivariable analyses

The confounders we included in the analyses were significantly related to all of the outcomes (stillbirths, neonatal mortality, perinatal mortality, preterm births, and very low birthweight) (data not shown) and to the reproductive history among teenagers (Table 1). In the logistic regression model adjusted for all confounders, infants of adolescents with a previous birth were at higher risk for perinatal mortality [OR = 2.35, CI 1.14, 4.86] and neonatal mortality [OR = 4.70, CI 1.60, 13.81]. Adolescents with a previous induced abortion had a higher risk for very low birthweight infants [OR = 2.74, CI 1.06, 7.09]. There were no significant differences in terms of preterm births and stillbirths related to obstetric history.

## Discussion

Using routinely collected perinatal data we examined the relationships between obstetric history and reproductive outcomes among adolescents. Compared to nulliparae, adolescents with a previous birth had a more than twofold higher risk for perinatal mortality and a more than fourfold higher risk for neonatal mortality.

The results of this study confirm the findings of studies with cross-sectional designs. For example, Hellerstedt et al. [8] found that the crude risk for neonatal deaths was 20 percent higher among multiparae compared to primiparous teenagers. Additionally, in the US, the risk for neonatal mortality was about 1.5-fold increased among 18–19 year old multiparae compared to primiparae of the same age [7]. Our study contradicts findings from a longitudinal US-study that found an almost twofold higher risk for perinatal deaths and a threefold higher risk for stillbirths among nulliparous women compared to adolescents with a previous birth [13]. However, the study was underpowered because of a small sample size and the results were not statistically significant. Other longitudinal studies also had limited sample sizes and could not examine rare outcomes such as perinatal mortality [11,12,14].

Due to the lack of studies on adolescent multiparae that considered confounders, the selection of potential confounders in this study derived from research on adolescent primiparae or adult women [18,20,21]. The confounders we examined did not explain the elevated risks for perinatal and neonatal mortality among adolescents with a previous birth compared to nulliparous teenagers. Rather, the adjustment for confounders strengthened the observed relationships. To inform future preventive efforts, further studies should attempt to identify the mediating factors that increase the risk among adolescents with a previous birth for neonatal and perinatal mortality.

Previous studies on the risks associated with a history of abortion among teenagers are sparse. Lao and Ho [22] found that a previous induced abortion among Hong Kong teenagers was not related to a higher risk for preterm birth. In our study, teenagers with a history of an abortion had a 3.4-fold higher risk for a stillbirth and a 2.2-fold higher risk for a preterm born infant than nulliparous adolescents. After adjustment for confounders these associations disappeared. In our study and in Hong Kong [22] teenagers with a previous abortion were characterized by a much higher smoking rate than the adolescent mothers with no abortion history. In the same group of teenagers, compared to nulliparous women, the risk for a very low birthweight infant was increased in the adjusted model. Because the confounders we examined (such as smoking during pregnancy or inadequate prenatal care) are related to both stillbirths [23] and very low birthweight [24] further studies are needed to understand this contradictory result.

In our sample, the rates of adverse outcomes largely correspond with Scottish data [10] but they were lower compared to the American studies of Blankson et al. (1993) [12] and Hellerstedt et al. (1995) [8], especially regarding preterm birth. However, neither the ethnic composition nor the social context of these US studies and our study can readily be compared. One reason for the lower incidence rates in preterm birth in Lower Saxony may be sought in the fact that the perinatal registry does not cover (planned and unplanned) out-of-hospital births. Also, the incidence rate of adolescents' pregnancies in Lower Saxony is slightly below the German average. This may reflect a less adverse environment compared to those areas with higher incidence rates such as Berlin and the eastern federal states, areas with higher unemployment rate, in particular among adolescents.

We have no information on known risk factors for adverse outcomes, especially of teenage pregnancies, such as domestic violence, stress, or poverty [20,25]. Smith and Pell compared the birth outcomes of primiparae and secundiparae between adolescent and adult mothers and found no differences among primiparae but higher perinatal mortality among adolescent than among adult secundiparae [10]. Smith and Pell concluded that a second birth during adolescence probably occurs more often in the context of poverty and poor nutrition than a second birth among mature women. Therefore, among teenagers living in a disadvantaged social context the accumulative burden of a second pregnancy may result in adverse outcomes. Our sample, however, is characterized by a vast proportion of migrant adolescents who usually are married and have access to strong support networks within their communities. The differences regarding the social context and behavioural characteristics (such as migrant status, lone motherhood, and smoking) among the three reproductive groups in our sample may point at the necessity for sociodemographically tailored approaches when attempting to improve the reproductive health of these women.

According to Klerman, findings from previous cross-sectional studies may be biased because they often compared any higher order births to nulliparous teenagers and missed important confounders [26]. The results of our study cannot readily be compared to these studies because we deleted adolescents who previously had a spontaneous miscarriage from the sample and thus only examined "true" nulliparae. Contrary to Hellerstedt et al. we compared the outcomes of the first birth to the outcomes of the second pregnancy while higher order pregnancies were excluded [8]. Additionally, we considered the outcome very low birthweight (< 1500 grams) instead of low birthweight (< 2500 grams) because the predictive value of the latter variable for children's health is still being debated [27]. We did not use intrauterine growth retardation as an outcome because the underlying growth norms refer to the German population and may not be valid for migrant newborns who account for more than one third in our sample. Previous studies on subsequent teenage pregnancy mostly have been from the US. However, the German adolescent population differs from the US population on several important aspects such as the ethnic composition. Also, contrary to the US, in Germany, prenatal care is free for all women regardless of their age, migration or employment status. Health insurance is mandatory. For refugees and for unemployed women the costs of prenatal care are covered by the community. Consistent with other studies from countries with free provision of prenatal care a huge proportion of teenagers in each reproductive group chose not to utilize this offer [28]. These adolescents may be characterized by a lower level of knowledge about the availability of prenatal care. Those who have had a previous birth or an abortion may anticipate negative comments on their condition by health care providers [28].

Our study has several limitations. To suggest causality this type of study has to be longitudinal. Adolescents who have a birth following a prior birth or an abortion are different in many ways from those who have a first birth with no previous pregnancies [26]. We have controlled for nationality, partner status, smoking, pre-pregnancy BMI and adequacy of prenatal care to differentiate between adolescents with different reproductive histories. However, we have no information on birth spacing. It may well be that a short inter-pregnancy interval is one of the underlying causes of worse outcomes among adolescents who had a previous pregnancy [29]. Intimate partner violence is another known risk factor for subsequent pregnancies during adolescence that we were not able to examine [30]. Alcohol is a known teratogenic substance that operates under a dose-response mechanism and drug use is associated with adverse pregnancy outcomes as well [31,32]. We could not access information on these substances. Further known risk factors for adverse pregnancy outcomes such as an unwanted pregnancy, stress, poverty, and vaginal infections also are not assessed in the routine perinatal survey. In summary, it is possible that our findings might be eliminated if we had accessed more confounders or if the study had a longitudinal design.

Although the rate of non-participating hospitals is rather small (2–13 percent), we cannot rule out a selection bias. Small numbers in some cells resulted in broad confidence intervals.

Induced abortions usually are recorded in the mother's passport but in the next pregnancy the women can choose to visit a new gynaecologist or midwife without bringing her mother's passport and thus deny the previous pregnancy. Therefore, underreporting of previous abortions is possible may have resulted in a classification bias.

A high proportion of teenagers who already gave birth to a child were characterized by Non-German nationality. Although we adjusted for nationality we cannot disregard that residual confounding may have occurred and that characteristics associated with migrant status might have affected the risk for adverse outcomes.

However, the current study expands on previous studies in several ways: it is not based on vital statistics but rather on data from a population sample prospectively collected by physicians and midwives. Thus, external validity and generalizability are satisfactory. Additionally, we incorporated several relevant confounders such as smoking in our analyses. Our study not only considers the adolescents' parity but draws attention explicitly to the reproductive history.

## Conclusion

We found among adolescents who already had a previous pregnancy a higher risk for the infant than among teenagers who reported to be pregnant for the first time even if relevant confounders are controlled. Among adolescents with a history of an abortion, the risks for stillbirth and preterm birth are increased but this can be explained by confounders. The prevention of the second pregnancy during adolescence is an important public health objective and should be addressed by health care providers who attend the first birth or the abortion and the follow-up care. Given the high proportion of migrant adolescent mothers in this sample, awareness of the cultural aspects of reproductive health is an important issue for researchers and health care workers to consider. Furthermore, health care workers should attempt to improve the pregnancy outcomes of subsequent teenage pregnancies by addressing modifiable risk factors, for example, supporting smoking cessation efforts. Studies that focus on examining the hypothesized mediators of social disadvantage, such as domestic violence, poverty, social support and educational opportunities, may also facilitate the development of effective interventions.

## Competing interests

The author(s) declare that they have no competing interests.

## Authors' contributions

BR analysed the data and mainly wrote the manuscript. BS assisted with interpreting the results and writing the manuscript. PW assisted with interpreting the results and writing the manuscript. The manuscript has been read and approved by all three authors.

## Pre-publication history

The pre-publication history for this paper can be accessed here:


